# Opposite Impact of REM Sleep on Neurobehavioral Functioning in Children with Common Psychiatric Disorders Compared to Typically Developing Children

**DOI:** 10.3389/fpsyg.2016.02059

**Published:** 2017-01-09

**Authors:** Roumen Kirov, Serge Brand, Tobias Banaschewski, Aribert Rothenberger

**Affiliations:** ^1^Institute of Neurobiology, Bulgarian Academy of SciencesSofia, Bulgaria; ^2^Center for Affective, Stress, and Sleep Disorders, Psychiatric Hospital of the University of BaselBasel, Switzerland; ^3^Clinic for Child and Adolescent Psychiatry, Central Institute of Mental HealthMannheim, Germany; ^4^Clinic for Child and Adolescent Psychiatry and Psychotherapy, University Medical Center GöttingenGöttingen, Germany

**Keywords:** REM sleep, neurobehavioral functioning, developmental psychopathologies, attention-deficit/hyperactivity disorder, tic disorder, co-morbidity

## Abstract

Rapid eye movement (REM) sleep has been shown to be related to many adaptive cognitive and behavioral functions. However, its precise functions are still elusive, particularly in developmental psychiatric disorders. The present study aims at investigating associations between polysomnographic (PSG) REM sleep measurements and neurobehavioral functions in children with common developmental psychiatric conditions compared to typically developing children (TDC). Twenty-four children with attention-deficit/hyperactivity disorder (ADHD), 21 with Tourette syndrome/tic disorder (TD), 21 with ADHD/TD comorbidity, and 22 TDC, matched for age and gender, underwent a two-night PSG, and their psychopathological scores and intelligence quotient (IQ) were assessed. Major PSG findings showed more REM sleep and shorter REM latency in the children with psychiatric disorders than in the TDC. Multiple regression analyses revealed that in groups with developmental psychopathology, REM sleep proportion correlated positively with scores of inattention and negatively with performance IQ. In contrast, in the group of TDC, REM sleep proportion correlated negatively with scores of inattention and positively with performance IQ. Whilst shorter REM latency was associated with greater inattention scores in children with psychopathology, no such an association existed in the group of TDC. Altogether, these results indicate an opposite impact of REM sleep on neurobehavioral functioning, related to presence or absence of developmental psychiatric disorders. Our findings suggest that during development, REM sleep functions may interact dissimilarly with different pathways of brain maturation.

## Introduction

Rapid eye movement (REM) sleep is characterized by bizarre dreaming consciousness upon lack of external input (Hobson, 2009; Nir and Tononi, 2010), which is accompanied by specific neurophysiologic signatures including wake-like low frequency desynchronized electroencephalogram (EEG) dominated by theta and gamma EEG oscillation, swift occurrence of REMs and pontine-geniculate-occipital waves and absence of muscle tone (Rechtschaffen and Kales, 1968; Hobson and Pace-Schott, 2002; Hobson et al., 2014). Remarkably, amongst all sleep-wake stages, REM sleep has a prominent role in enabling neuronal plasticity, increased synaptic connectivity, and immediate early genes synthesis (Ribeiro et al., 2002; Grosmark et al., 2012) and is signified by a strong cortical activation (De Gennaro et al., 2004; Massimini et al., 2010). Thus, the functional roles of REM sleep and dreaming have been of sustained interest. In healthy adults, REM sleep has been shown to support many adaptive functions. These include consolidation of emotional memory (Wagner et al., 2001; Nishida et al., 2009), resolution of affect (van der Helm et al., 2011), further transformation of previously consolidated during non-REM (NREM) sleep memories (Walker and Stickgold, 2010; Rasch and Born, 2013; Llewellyn and Hobson, 2015), consolidation of procedural or implicit memory and motor learning (Yordanova et al., 2008; Diekelmann and Born, 2010), and promoting human heuristic creativity (Cai et al., 2009; Brand et al., 2010). REM sleep’s physiological and psychological features also have been associated with more complex functions. For example, REM sleep has been suggested to heighten autobiographic memory (Horton and Malinowski, 2015; Malinowski and Horton, 2015) and to render previously encoded memories more distinct through its hyper-associative dreaming state (Llewellyn, 2013; Llewellyn and Hobson, 2015). Also, it is thought to incorporate these previously encoded memories into a broader vital context, thus embedding them in consolidated residuals of hypotheses, emotions, basic needs, and individual genetic traits (Kirov, 2013). Next, REM sleep has been proposed to generate an innate virtual model of the world, thus modulating predictive coding (Hobson and Friston, 2012; Hobson et al., 2014; Hopkins, 2016). In view of REM sleep deviations in almost all psychiatric conditions (Benca et al., 1997; Gottesmann and Gottesman, 2007; Baglioni et al., 2016), REM sleep also has been regarded as a mechanism mediating brain adaptation in normal and pathological conditions (Benca et al., 1992; Horne, 2013, 2015; Goldstein and Walker, 2014; Hobson et al., 2014; Hopkins, 2016; Mota et al., 2016).

From a developmental perspective, infants have much more REM sleep quantity, which descends through childhood and adolescence, than adults (Roffwarg et al., 1966; Brand and Kirov, 2011). It has been proposed that this developmental decrease in REM sleep sub-serves brain maturation through synaptic reorganization and/or pruning, internally generated stimulation of neuronal assembles, or genetic programming (Marks et al., 1995; Jouvet, 1998; Feinberg and Campbell, 2010; Hopkins, 2016). In this regard, an insufficient decline or variations of the normal REM sleep decline during development is proposed to underpin a broad spectrum of child and adolescent psychiatric disorders (Partonen, 1998; Kobayashi et al., 2004; Garcia-Rill et al., 2008; Brand and Kirov, 2011; Kirov and Brand, 2014). Given that both the hypothalamus-pituitary-adrenocortical axis activation and REM sleep overdrive are closely associated in psychiatric conditions (Steiger et al., 2013), this notion has received an indirect support by documenting an existence of elevated cortisol levels in association with disturbed sleep and impaired neurobehavioral functions in a cohort of children with various psychiatric symptoms (Hatzinger et al., 2012), and thus, probably, with deviant stress sensitivity (Brand and Kirov, 2011; Gruber, 2014). However, whether and how REM sleep in common developmental child psychiatric disorders may be linked to neurobehavioral functioning is still less well understood.

We have shown previously a REM sleep overdrive in children with attention-deficit/hyperactivity disorder (ADHD), Tourette syndrome/chronic tic disorder (TD) and ADHD/TD comorbidity, with this REM sleep overdrive being associated mostly with ADHD core symptoms (Kirov et al., 2007). More recently, we have demonstrated the following pattern of associations between REM sleep quantity and neurobehavioral functions in youth ADHD: (1) In children with ADHD, REM sleep proportion correlated positively with inattention and negatively with performance intelligence quotient (IQ). (2) In opposition, the proportion of REM sleep in typically developing children (TDC) correlated negatively with inattention and positively with performance IQ (Kirov et al., 2011). Similarly, another recent study showed that whereas in youths with ADHD, theta (4–8 Hz) EEG power during REM sleep correlated negatively with emotional memory consolidation, in healthy individuals, this correlation was positive (Prehn-Kristensen et al., 2013). Collectively, these latter findings suggest at least a bi-directional role of REM sleep and its physiology, depending on presence or absence of ADHD psychopathology.

The present study aimed at further investigating the impact of REM sleep on neurobehavioral functioning in children with a broader spectrum of common developmental psychiatric disorders. In an attempt to clarify if the associations between REM sleep proportion and neurobehavioral functions reported previously (Kirov et al., 2011) were only linked to ADHD psychopathology possibly reflecting a disorder-specific dysfunction of neural regulation leading to both daily symptoms expression and sleep disturbances (Kirov et al., 2007), we enrolled in the present study larger sample sizes of children with ADHD, TD and ADHD/TD comorbidity and compared them to healthy TDC, while testing associations not only between REM sleep parameters and psychopathological scores, but also considering children’s IQ. We hypothesized a differential impact of REM sleep on cognitive and behavioral functioning in the children with the continuum of common developmental psychiatric disorders (Gaze et al., 2006; Kirov et al., 2011) compared to the TDC. Also, we proposed that REM sleep may be associated dissimilarly with different psychopathological scores across the children’s specific psychiatric diagnoses.

## Materials and Methods

### Subjects

Eighty-eight children aged between 8 and 16 years (66 outpatients with ADHD, TD and ADHD/TD comorbidity and 22 TDC) participated in the study. All children and their parents were native German speakers. Children were examined clinically by two independent board-certified child psychiatrists and underwent clinical tests for neurological and internal diseases, including routine EEG and electrocardiogram. All patients were consecutive referrals to the Clinic for Child and Adolescent Psychiatry at the University Medical Center of Goettingen, Germany. They were diagnosed according to the Diagnostic and Statistical Manual of Mental Disorders 4th edition (DSM-IV; American Psychiatric Association [APA], 1994) with ADHD-combined subtype (314.01), Tourette syndrome/chronic TD (307.22/307.23) and ADHD/TD comorbidity. TDC were recruited among friends and relatives of the clinical staff. Exclusion criteria for the children with psychopathology were presence of internal diseases, neurological or psychiatric problems not associated with ADHD and TD, and verbal, performance and total IQ < 70 (the German version of Wechsler Intelligence scale for children; Tewes, 1999), as evaluated by the certified child psychiatrists. Further, the exclusion criteria applied for the controls were neuropsychiatric or internal diseases, IQ < 70 and current sleep problems, as assessed during an adaptation night with polysomnography (PSG). None of the patients had clinically expressed psychiatric disorders different from ADHD, TD and ADHD/TD co-morbidity, somatic and neurological diseases and IQ < 70, and none of the TDC had neuropsychiatric or internal diseases, IQ < 70 and current sleep problems.

The 88 children formed the following four groups. Twenty-four children (27.3%) with ADHD-combined subtype, 21 (23.9%) with TD, 21 (23.9%) with ADHD/TD comorbidity, and 22 (25%) TDC (**Table 1**). The four groups were matched for age and gender, but not for IQ. However, as can be seen in **Table 1**, the groups did not differ significantly for IQ.

**Table 1:** Demographic and clinical characteristics of the groups.

Group	ADHD (*n* = 24)	TD (*n* = 21)	ADHD/TD (*n* = 21)	Controls (*n* = 22)
	Means (*SD*)	Means (*SD*)	Means (*SD*)	Means (*SD*)
Age (months)	134.25 (24.56)	141.52 (21.37)	132.43 (27.90)	137.14 (27.41)
Age (years)	11.19 (2.05)	11.79 (1.78)	11.04 (2.32)	11.42 (2.28)
Age range (years)	8.0–15.0	8.0–15.8	8.0–16.0	8.0–15.6
Gender (male:female)	20:4	19:2	19:2	19:3

Symptom onset (years)	5.8–6.7	5.9–6.9	5.8–6.9	N/A
MED:NMED	10:14	9:12	7:14	N/A

**Intelligence (IQ)**				
Verbal	103.33 (11.08)	100.67 (11.93)	100.56 (10.57)	105.68 (9.06)
Performance	101.98 (10.08)	100.67 (12.59)	100.47 (11.77)	104.50 (10.49)
Total	102.71 (10.67)	101.57 (11.50)	101.88 (10.58)	106.77 (10.43)

CPRS (10-item score)	18.00 (6.67)	9.67 (5.41)^d^	17.71 (4.13)	3.55 (2.70)^a,b,c^
**CBCL (*T*-scores)**				
Attention problems	66.04 (11.46)	61.67 (6.80)^d^	70.86 (10.39)	51.73 (1.51)^a,b,c^
Internalizing problems	63.17 (13.00)	61.86 (9.27)	62.90 (9.02)	46.00 (6.86)^a,b,c^
Externalizing problems	64.04 (11.71)	61.43 (10.95)	66.67 (10.71)	42.77 (8.01)^a,b,c^
Aggressive behavior	67.21 (67.2)	55.90 (8.37)^d^	69.29 (10.89)	50.64 (1.62)^a,b,c^
Delinquent behavior	63.42 (10.59)	55.38 (7.52)^d^	62.90 (8.24)	51.64 (3.18)^a,b,c^
Total	65.17 (10.74)	59.86 (9.27)^d^	67.43 (8.09)	47.64 (8.24)^a,b,c^
**LOI**				
“Yes” answers	9.83 (6.79)	10.38 (4.69)	10.48 (7.17)	8.64 (4.47)
Resistance score	10.54 (7.41)	13.95 (5.19)^d^	11.14 (6.68)	8.73 (6.31)^b,c^
Interference score	9.29 (6.36)	14.95 (9.41)^d^	11.19 (15.89)	7.23 (5.37)^b,c^

**TSSS**	N/A	3.41 (1.47)	4.17 (1.72)	N/A

**PSG clock-times**				
Lights off	20:05–22:50	20:25–23:20	20:15–23:00	20:25–22.50
Lights on	8:30–9:29	7:45–9:05	8:10–9:15	8:15–9:10

*ADHD, attention-deficit/hyperactivity disorder; TD, tic disorder; ADHD/TD, co-morbidity; MED, medicated before study; NMED, never medicated; N/A, not applicable; CPRS, Conner’s parent rating scale; CBCL, child behavior checklist; LOI, Leyton obsession inventory; TSSS, Tourette syndrome severity scale; PSG, polysomnography. Significant (*p* < 0.05) group differences (independent samples *t*-tests): ^a^ADHD vs. Controls; ^b^TD vs. Controls; ^c^ADHD+TD vs. Controls; ^d^TD vs. ADHD and ADHD/TD.*

Most patients (*n* = 40; 60.6%) have never received any medications. The medication of the others (*n* = 26; 39.4%) was as follows: (1) Nine boys and one girl with ADHD were treated with Methylphenidate Hydrochloride (MPH: Ritalin^®^, Novartis Pharma GmbH, Nuremberg, Germany). (2) In the TD group, seven boys were treated with Tiaprid (Tiaprid^®^, neuraxpharm Arzneimittel GmbH, Langenfeld, Germany), and two boys with Haloperidol (Haloperidol^®^; ratiopharm direct GmbH, Ulm, Germany). (3) Six boys with ADHD/TD comorbidity received a combination of MPH and Haloperidol, and one girl received Tiaprid (**Table 1**). The medication of the 26 children with psychiatric disorders was discontinued 5 to 14 days before study.

The study was performed according to the clinical standards of the Declaration of Helsinki and approved by the Local Ethics Committee at the University Medical Center of Goettingen, Germany. A detailed description of the investigation was provided to the parents and their children. Parents of each child signed written consent and children gave age-appropriate consent.

### Psychometric Assessment

To further provide detailed data for a quantitative assessment of psychopathological problems across groups, control and patient groups were carefully assessed by means of psychometric questionnaires, including Child Behavior Checklist (CBCL; Achenbach and Edelbrock, 1983), Conners Parent Rating Scale (CPRS; Goyette et al., 1978) and Leyton Obsessional Inventory (LOI; Berg et al., 1986). Only for the children with TD and ADHD/TD comorbidity, the Tourette Syndrome Severity Scale (TSSS; Shapiro et al., 1988) was used.

Child Behavior Checklist items, scored on a 3-point Likert scale ranging from not true to often true, were used for defining child behavior problems and assessing childhood psychopathology empirically. Items were combined to yield various narrower band scales: (1) Attention problems: nine items (Cronbach’s α = 0.89). (2) Internalizing problems: seven items (Cronbach’s α = 0.80). Externalizing problems: 17 items (Cronbach’s α = 0.91). Aggressive behavior: 13 items (Cronbach’s α = 0.90). Delinquent behavior: 11 items (Cronbach’s α = 0.89). Total CBCL score: 57 items (Cronbach’s α = 0.91).

For a quantitative assessment of the level of hyperactivity and impulsiveness, the short 10-item version of CPRS (3-point scale ranging from not true to often true) was used (Cronbach’s α = 0.89).

On a 20-item LOI concerning obsessive-compulsive behavior, children were asked to respond in the items with ‘yes’ or ‘no’ scored as 1 or 0 points, respectively. When ‘yes’ responses were obtained, children were assessed for either resistance to their symptoms or interference with other activities that the symptoms cause by 4-point scales (Cronbach’s α = 0.90).

Tourette Syndrome Severity Scale (five items) was applied to quantitatively measure the severity of motor and vocal tics (rated by ‘not true’, ‘somewhat’, and ‘often true’ and scored as 0, 1, and 2 points, respectively (Cronbach’s α = 0.86).

Child Behavior Checklist and CPRS scores were rated by the mothers of the children, whereas the scores of LOI and TSSS were rated by experts after psychiatric interviews with mothers and children. As shown in **Table 1**, the groups significantly differed in each CBCL subscale, CPRS and LOI subscales, with no significant differences between TD and ADHD/TD comorbidity groups on TSSS. All psychometric and IQ evaluations of the patient and control groups of children were made 1 or 2 days before conducting a two-night PSG.

### Polysomnographic (PSG)

All children underwent a PSG in the sleep laboratory during two consecutive nights. An unrestrained sleep regime was employed with the major goal to avoid as much as possible situational variations that could potentially affect sleep findings. The PSG included EEG (C3 and C4) electrodes referenced to the right (A2) and left (A1) mastoids, electrooculogram recorded from electrodes above and below the right eye and the outer canthi of the orbits and submental electromyogram. All PSG recordings were performed on a 21-channel polyphysiograph (Nihon Kohden, Tokyo, Japan) with electrode impedance <5 kohms and stored on a computerized video-monitoring system (Sagura Polysomnograph 2000, Sagura Medizintechnik GmbH, Muhlheim, Germany). PSG data were analyzed visually in 30-s epochs according to standard criteria (Rechtschaffen and Kales, 1968) by three independent certified technicians blind to subject grouping: inter-rater agreement [>89%; Cohen’s kappa: (range 0.89–0.93)]. The first PSG night served as an adaptation night, during which a monitoring for presence of primary sleep disorders was conducted. To avoid the first-night effect, sleep PSG data were taken only from the second night (Kirov et al., 2012).

### Statistics

Data were analyzed using IBM SPSS Statistics 19 (IBM Corp., Armonk NY, USA). All demographic (except gender), IQ and psychometric data and all sleep PSG data were first tested for normality of distribution by means of Kolmogorov–Smirnov test, with the following results obtained: (1) patients (*n* = 66: *Z* < 1.28, *p* > 0.12), (2) TDC (*n* = 22: *Z* < 1.19, *p* > 0.14), (3) ADHD group (*n* = 24: *Z* < 1.11, *p* > 0.16), (4) TD group (*n* = 21: *Z* < 1.01, *p* > 0.18), and (5) ADHD/TD comorbidity group (*n* = 21: *Z* < 1.22, *p* > 0.12), verifying a Gaussian distribution for all data sets. Therefore, parametric statistics was used. The demographic and clinical data, excluding gender (chi-squared test) were statistically evaluated by means of independent samples *t*-tests (**Table 1**). All sleep PSG parameters were subjected to a one-way multivariate analysis of variance (MANOVA) with one between-subjects factor group. In case of significant group effects, independent samples *t*-tests were conducted. The alpha level of significance was fixed at 0.05.

To control for presence of a possible rebound effect on sleep due to medication ceasing, independent samples *t*-tests were conducted to compare the PSG parameters between (1) ADHD patients medicated (*n* = 10) and unmedicated (*n* = 14), (2) TD patients medicated (*n* = 9) and unmedicated (*n* = 12), (3) ADHD/TD comorbidity patients medicated (*n* = 7) and unmedicatd (*n* = 14) before study.

To test if any index of the neurobehavioral functioning (psychometric and IQ scores) may be a specific determinant of REM sleep parameters in the psychopathological group (ADHD, TD and ADHD/TD comorbidity; *n* = 66), multiple regression stepwise analyses were conducted, where in separate analyses different REM sleep parameters were included as dependent variables, and psychometric and IQ scores, group, age (in months), gender, and medication status (**Table 1**) were used as independent predictors. The same multiple regression stepwise analyses were also conducted for the TDC, as well as separately in the three patient groups. Finally, to test whether other sleep PSG variables may be associated with the neurobehavioral functioning across groups, the multiple regression stepwise analyses described above were performed additionally.

## Results

### Major PSG Findings

Multivariate analysis of variance results showed significant group effects on sleep onset latency (SOL; *F*_3/84_ = 3.12, *p* = 0.03), slow-wave sleep (SWS) latency (*F*_3/84_ = 2.96, *p* = 0.04), REM sleep latencies taken from lights-off (*F*_3/84_ = 3.06, *p* = 0.03) and from SOL (*F*_3/84_ = 3.36, *p* = 0.02), and the absolute (in minutes: *F*_3/84_ = 5.56, *p* = 0.002) and the relative (% of total sleep time: *F*_3/84_ = 5.40, *p* = 0.002) REM sleep amounts. *Post hoc* independent samples *t*-tests revealed: (1) A longer SOL in the TD group compared to the ADHD and TDC groups (*t*_41(43)_ > 2.15, *p* < 0.04). (2) A prolonged SWS latency in the TD group compared to the ADHD and the TDC (*t*_41(43)_ > 1.99, *p* < 0.05). (3) Shorter REM sleep crude (taken from lights-off) and adjusted to SOL latencies (*t*_41(44)_ > 2.63, *p* < 0.01) and greater absolute and relative amounts of REM sleep (*t*_41(44)_ > 2.99, *p* < 0.005) in all the three groups with psychiatric disorders relative to controls (**Table 2**). None of the REM sleep parameters differed significantly across the groups of ADHD, TD and ADHD/TD comorbidity (*t*_40(43)_ < 1.48, *p* > 0.09; **Table 2**). No significant rebound effects on either PSG parameters due to medication ceasing for the ADHD (*t*_22_ < 1.10, *p* > 0.28), the TD (*t*_19_ < 1.05, *p* > 0.31) and the ADHD/TD comorbidity (*t*_19_ < 1.33, *p* > 0.20) patients were found, verifying thus no effects of medication ceasing on the PSG findings, as detailed in **Table 2**.

**Table 2:** Sleep PSG parameters: results from independent samples *t*-tests.

Group	ADHD (*n* = 24)	TD (*n* = 21)	ADHD/TD (*n* = 21)	Controls (*n* = 22)
	Means (*SD*)	Means (*SD*)	Means (*SD*)	Means (*SD*)
**PSG parameters**				

Time in bed (TIB)	598.31 (72.96)	582.21 (73.97)	607.19 (55.82)	565.66 (46.01)
Total sleep time (TST)	555.98 (69.85)	528.21 (79.03)	555.67 (48.28)	525.09 (40.89)
Sleep onset latency (SOL)	15.79 (13.81)	27.86 (23.29)^d^	19.90 (19.76)	12.91 (11.19)^b^
SE (TST % of TIB)	92.95 (3.04)	90.58 (5.31)	91.68 (4.64)	92.91 (3.25)

**Sleep stages latencies**				

Slow-wave sleep (SWS)	30.42 (14.94)	45.33 (19.40)	31.43 (20.12)	25.95 (13.33)^b^
REM sleep (crude)	117.40 (38.50)	123.29 (44.96)	121.02 (47.46)	146.00 (42.96)T^a,b,c^
REM sleep (adjusted)	101.58 (35.81)	105.38 (38.69)	102.57 (44.53)	133.09 (35.05)^a,b,c^

**Sleep stages duration (in min and as % of TST)**				
Wake (min)	15.32 (10.37)	14.86 (12.56)	19.47 (19.80)	12.76 (14.24)
Stage 1 (min)	15.49 (11.32)	21.25 (17.07)	17.15 (19.01)	22.46 (15.12)
Stage 2 (min)	245.05 (39.27)	230.97 (49.48)	245.31 (37.67)	244.13 (34.07)
SWS (min)	128.64 (36.74)	120.51 (35.31)	117.46 (35.15)	124.26 (29.32)
REM sleep (min)	143.55 (36.30)	131.84 (23.28)	147.68 (34.86)	114.30 (20.38)^a,b,c^
Movement time (min)	8.36 (3.88)	8.85 (4.02)	7.11 (3.30)	8.74 (3.45)
Wake (% of TST)	2.79 (1.97)	2.81 (2.33)	3.51 (3.68)	2.37 (2.59)
Stage 1 (% of TST)	2.79 (2.04)	4.00 (3.07)	3.11 (3.47)	4.23 (2.76)
Stage 2 (% of TST)	44.08 (4.55)	43.57 (5.88)	44.08 (5.05)	46.44 (4.64)
SWS (% of TST)	23.22 (5.91)	22.98 (5.94)	21.28 (6.43)	23.78 (5.66)
REM sleep (% of TST)	25.70 (4.56)	25.00 (3.04)	26.47 (4.68)	21.83 (3.84)^a,b,c^
Movement time (% of TST)	1.49 (0.61)	1.65 (0.65)	1.27 (0.60)	1.66 (0.43)

*ADHD, attention-deficit/hyperactivity disorder; TD, tic disorder; ADHD/TD, co-morbidity; PSG, polysomography; SE, sleep efficiency; REM, rapid eye movement; REM sleep latency was taken from lights-off (crude) and from sleep onset latency (adjusted). SWS included Stage 3 non-REM sleep and Stage 4 of non-REM sleep. Significant between-group differences (*p* < 0.05): ^a^ADHD vs. Controls; ^b^TD vs. Controls; ^c^ADHD/TD vs. Controls; ^d^TD vs. ADHD.*

### Multiple Regression Analyses

**FIGURE 1:**
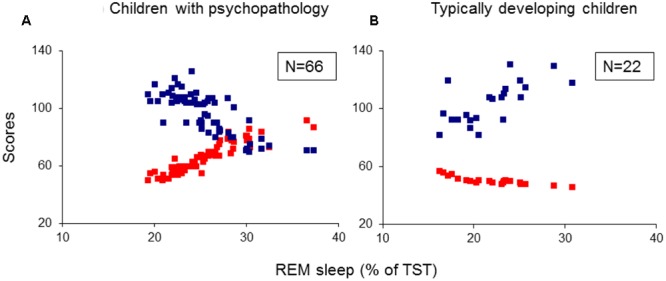
**Multiple regression analysis: correlations of relative REM sleep proportion with scores of inattention and performance IQ. (A)** Shows the correlations of relative REM sleep proportion with scores of inattention and performance IQ in the group of children with psychopathology (*N* = 66); **(B)** Shows the correlations of relative REM sleep proportion with scores of inattention and performance IQ in the group of typically developing children (*N* = 22). REM, rapid eye movement; IQ, intelligence quotient; Red squares: individual values of inattention scores; Blue squares: individual values of performance IQ.

**FIGURE 2:**
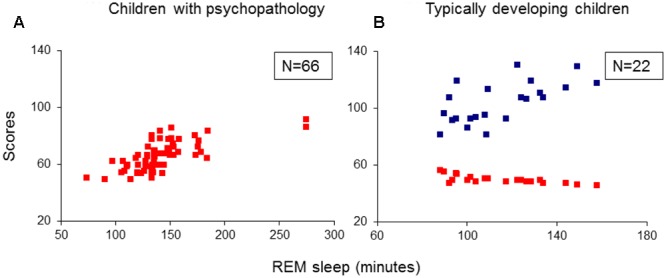
**Multiple regression analysis: correlations of absolute REM sleep amount with scores of inattention and performance IQ. (A)** Shows the correlations of absolute REM sleep amount with scores of inattention in the group of children with psychopathology (*N* = 66); **(B)** Shows correlations of absolute REM sleep amount with scores of inattention and performance IQ in the group of typically developing children (*N* = 22). REM, rapid eye movement; IQ, intelligence quotient; Red squares: individual values of inattention scores; Blue squares: individual values of performance IQ.

For each analysis, Durbin–Watson statistics showed that residuals of the independent predictors were independent (*d* values: 1.5–2.3; Durbin and Watson, 1951). In all children with psychiatric disorders (ADHD, TD and ADHD/TD comorbidity: *n* = 66), the relative proportion of REM sleep was predicted independently by inattention (CBCL) and performance IQ (*R* = 0.907; *R*^2^ = 0.824; Adjusted *R*^2^ = 0.818; *F*_2/63_ = 147.02, *p* < 0.001). Whereas the REM sleep proportion correlated positively with scores of inattention (*B* = 0.286; β = 0.704; *t* = 8.60, *p* < 0.001), it correlated negatively with performance IQ (*B* = -0.070; β = -0.248; *t* = -3.03, *p* = 0.004; **Figure 1A**). In the group of TDC (*n* = 22), the relative REM sleep proportion also was predicted independently by inattention and performance IQ (*R* = 0.820; *R*^2^ = 0.672; Adjusted *R*^2^ = 0.637; *F*_2/19_ = 19.44, *p* < 0.001). In contrast to the children with psychopathologies, however, in the TDC, REM sleep proportion correlated negatively with the inattention (*B* = -1.35; β = -0.532; *t* = -3.57, *p* = 0.002) and positively with the performance IQ (*B* = 0.112; β = 0.422; *t* = 2.83, *p* = 0.01; **Figure 1B**). These results were partially supported by an additional multiple regression analyses, where the absolute (in minutes) amount of REM sleep was a dependent variable. In the 66 children with psychopathologies, the absolute REM sleep proportion was only predicted by, and correlated positively with inattention (*R* = 0.693; *R*^2^ = 0.480; Adjusted *R*^2^ = 0.472; *F*_1/64_ = 59.13, *p* < 0.001; *B* = 2.19; β = 0.693; *t* = 7.69, *p* < 0.001; **Figure 2A**). In the TDC, the absolute REM sleep amount was predicted independently by performance IQ and inattention (*R* = 0.723; *R*^2^ = 0.523; Adjusted *R*^2^ = 0.473; *F*_2/19_ = 10.41, *p* = 0.001). It correlated positively with performance IQ (*B* = 0.646; β = 0.459; *t* = 2.56, *p* = 0.02) and negatively with inattention (*B* = -5.14; β = -0.383; *t* = -2.13, *p* = 0.04; **Figure 2B**).

**FIGURE 3:**
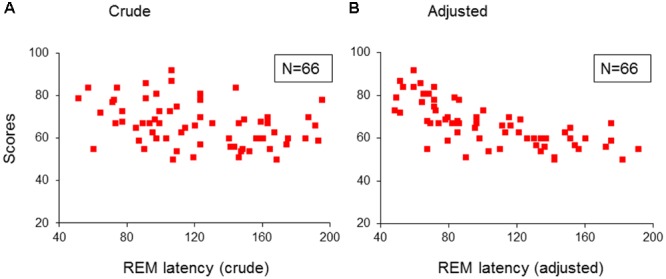
**Multiple regression analysis: correlations of REM sleep latencies with scores of inattention in children with psychopathology. (A)** Shows the correlations of crude REM sleep latency with scores of inattention in the group of children with psychopathology (*N* = 66); **(B)** Shows the correlations of adjusted REM sleep latency with scores of inattention in the group children with psychopathology (*N* = 66). REM, rapid eye movement; Crude: REM sleep latency taken from lights-off; Adjusted: REM sleep latency taken from sleep onset. Red squares: individual values of inattention scores.

Further, both the crude (taken from lights-off) and the adjusted (taken from sleep onset) REM sleep latencies in the 66 children with psychopathologies, were only predicted by, and correlated negatively with inattention (*R* > 0.357; *R*^2^ > 0.127; Adjusted *R*^2^ > 0.114; *F*_1/64_ > 9.33; *p* < 0.03; *B* = -1.36/-2.66; β = -0.357/-0.696; *t* = -3.05/-7.76, *p* = 0.003/<0.001; **Figures 3A/3B**). No model extracted any predictors for these REM sleep latencies in the TDC.

When applied the multiple regression analyses, where the relative and absolute amounts of REM sleep and REM latencies were dependent variables to each psychopathological group separately, and the following patterns of results were obtained: (1) In the ADHD group, the relative REM sleep proportion was predicted independently by inattention and performance IQ, and correlated positively with inattention and negatively with performance IQ. (2) In the TD group, the relative REM sleep proportion was predicted independently by inattention and interference LOI scores, and correlated positively with scores of inattention and LOI (interference scores). (3) In the group of ADHD/TD comorbidity, the relative REM sleep proportion correlated positively with inattention (**Table 3**). In each one of the psychopathological groups separately, the absolute REM sleep amount correlated positively with only inattention (*R* > 0.580; *R*^2^ > 0.337; Adjusted *R*^2^ > 0.302; *F*_1/19(22)_ > 9.65, *p* < 0.001; *B* > 1.98; β > 0.580: *t* > 3.01, *p* < 0.006). Further, in each one of the groups with psychopathologies, both the crude and the adjusted REM sleep latencies correlated negatively with only inattention (*R* > 0.441; *R*^2^ > 0.194; Adjusted *R*^2^ > 0.152; *F*_1/19(22)_ > 4.59, *p* < 0.04; *B* > -2.56; β > -0.441; *t* > -2.14; *p* < 0.04).

**Table 3:** Predictors of relative (% of TST) REM sleep proportion in the groups of ADHD, TD and ADHD/TD comorbidity.

Group	Predictors	*R*	*R*^2^	Adj. R^2^	*F* (d/f)	*p*	*B*	β	*t*	*p*
ADHD	Inattention				67.69		0.214	0.537	3.22	0.004
(*n* = 24)	Performance IQ	0.920	0.846	0.831	(2/21)	<0.001	-0.127	-0.417	-2.49	0.02

TD	Inattention				124.84		0.309	0.690	87.75	<0.001
(*n* = 21)	LOI (interference)	0.966	0.933	0.925	(2/18)	<0.001	0.119	0.368	4.66	0.008

ADHD/TD										
comorbidity					117.49					
(*n* = 21)	Inattention	0.928	0.861	0.853	(1/19)	<0.001	1.98	0.928	10.84	<0.001

*ADHD, attention-deficit/hyperactivity disorder; TD, tic disorder; TST, total sleep time; REM, rapid eye movement; IQ, intelligence quotient; LOI, Leyton Obsessional Inventory.*

Additional multiple regression analyses did not extract any predictors for SOL, SWS latency, as well as for the other sleep stages proportion and PSG parameters, including total sleep time and sleep efficiency.

## Discussion

The key findings of the present study were that among a sample of children diagnosed with ADHD, TD and ADHD/TD comorbidity, the more REM sleep amount was associated with higher scores of inattention and lower scores of performance IQ. By contrast, in TDC, the more REM sleep amount was associated with lower scores of inattention and higher performance IQ. Further, whereas in children with psychiatric disorders, the shorter REM sleep latencies were associated with higher scores of inattention, no such association was found for the TDC. The present findings add to the current literature in that we observed “double-edged” associations between REM sleep PSG parameters and neurobehavioral functioning, not only in ADHD (Kirov et al., 2011; Prehn-Kristensen et al., 2013). Notably, this study clearly showed that this opposite impact of REM sleep on daytime neurobehavioral functions is also observable in a broader spectrum of developmental child psychiatric conditions, as compared with TDC. Collectively, these findings give support to our hypothesis that REM sleep parameters may be associated dissimilarly with daytime neurobehavioral functions, depending on presence or absence of developmental psychiatric disorders. Although the relative REM sleep proportion in each of the three psychopathological groups was predicted consistently by inattention, some observations (**Table 3**) merit further attention. First, the observed positive association between REM sleep proportion and interference LOI scores in the TD group might underline the closeness of TD with subclinical obsessive-compulsive symptoms. Second, the modest differences in REM sleep predictors across the groups (**Table 3** and the reported within text results) may be accounted for by variations in statistical power due to the relatively small sample sizes included in each group.

The present data do not allow a deeper introspection into the exact neurobiological and psychological mechanisms underlying the pattern of associations, as found and described above. It is notable, however, that currently reported REM sleep alterations in children with a broad spectrum of psychiatric disorders correspond to findings in adults where psychiatric conditions such as depression, major depressive disorder, and post-traumatic stress disorder are featured by enhanced amounts of REM sleep (Benca et al., 1997; Wang et al., 2015; Baglioni et al., 2016). In adults, two parallel ways with a common source in the monoaminergic or cholinergic systems have been assumed to regulate both REM sleep and symptoms of depression (Wang et al., 2015). Likewise, we have proposed previously that altered aminergic-cholinergic ratio may lead to a REM sleep overdrive in the spectrum of developmental psychiatric conditions, as shown in the present study (Kirov et al., 2004, 2014; Brand and Kirov, 2011; Kirov and Brand, 2014). Further, specific brain regions including the hippocampus, amygdala and medial pre-frontal cortex may contribute to both daily symptoms and REM sleep impairments in both depressive adults (Wang et al., 2015) and children with ADHD (Hart et al., 2013) While the precise neurobiological mechanisms of the co-existing deviations in daily behaviors and REM sleep remain to be established (Wang et al., 2015), our current findings on the relationships between REM sleep amount and daily behavior both in patients and healthy children may open a relevant new line of understanding by considering the contra-directionality of these relationships. If increased REM sleep amounts in children were expressions of co-impaired regulation of REM sleep and attention, increased REM sleep might not predict superior achievements in TDC. If, on the contrary, increased REM sleep in patients reflected compensation, it should have predicted less symptoms severity in patients. The currently found bi-directionality of associations suggests that functional efficiency of REM sleep might be critically impaired in children with psychiatric disorders. There is evidence that the functional efficiency of REM sleep may vary both in pathological and normal conditions. For example, chronic stress in rats has been found to synchronize the theta rhythm between the hippocampus and amygdala, which was accompanied by increased amounts of REM sleep (Hegde et al., 2011). Further, Pellicciari et al. (2013) provide evidence for functional inefficiency of REM sleep alpha activity in depression in humans, which can be remediated by repetitive trans-cranial magnetic stimulation. These observations imply that the functional rhythms and associated mechanisms during REM may be primarily impaired in children with psychiatric disorders. Hence, in pathology, the increased REM sleep amount may reflect an attempt to compensate for functional inefficiency. While functional inefficiency of REM sleep may not be compensated by increased REM sleep amount in patients, increase in functionally efficient REM sleep in TDC is associated with an improved attention and higher performance IQ.

The present results also imply effects on psychological functioning. REM sleep plays a role in the consolidation of negative emotional memories (Wagner et al., 2001; Nishida et al., 2009; Goldstein and Walker, 2014) and resolution of affect through dissipation of amygdala activity in response to previous emotional experiences, thus reducing next-day subjective emotionality (van der Helm et al., 2011). In this regard, the present results suggest that those REM sleep neuronal mechanisms sub-serving successful emotional processing may be intact in the TDC and may be insufficient or impaired in the children with the spectrum of psychiatric disorders. Thus, our results point tentatively to a possible contribution of emotional liability and related anxiety in these child psychiatric disorders (Banaschewski et al., 2012; Gruber, 2014), Since a wealth of empirical evidence show that children with common developmental psychopathologies display greater difficulties in coping with emotional problems, inappropriate behaviors and social interactions (Swain et al., 2007; Brand and Kirov, 2011; Kirov and Brand, 2014; Kirov et al., 2014), this may affect the natural functions of REM sleep and may lead to impaired attention and procedural skills, respectively. Further, from a view point of the aminergic-cholinergic reciprocal interaction model for NREM-REM sleep cycle regulation (Hobson et al., 1975), we have proposed previously that altered aminergic-cholinergic ratio may lead to a REM sleep overdrive in the spectrum of developmental psychiatric conditions, as shown in the present study (Kirov et al., 2004, 2014; Brand and Kirov, 2011; Kirov and Brand, 2014). Though speculative, changes in this ratio may lead to dissimilar functions of REM sleep for implicit motor memory. Indirect support to this assumption comes from two consecutive studies. Whilst suppression of REM sleep induced by noradrenergic agonists led to a slight improvement of procedural memory, suppressing REM sleep by application of the cholinergic antagonists have produced opposite effects (Rasch et al., 2009a,b). Thus, REM sleep physiological, neurochemical and psychological features may interact differently with pathological traits in psychiatric conditions and, on the other hand, in normative behavioral functions in healthy youths.

Our findings are in accord with previous research, observing an adverse impact of REM sleep overdrive or its EEG signatures on daytime neurobehavioral functions in children with ADHD compared to TDC (Kirov et al., 2007, 2011; Prehn-Kristensen et al., 2013). Yet, they are at odds with findings from other studies. An earlier study has found a less REM sleep proportion and prolonged REM sleep latency in a community-based sample of 5- to7-years-old children with ADHD relative to controls, and has demonstrated that the less REM sleep and the longer REM latency correlated positively with inattention and hyperactivity/impulsivity (O’Brien et al., 2003). Another study conducted among 6- to 16-years-old children with TD, ADHD and TD/ADHD comorbidity, diagnosed according to DSM-III-R (American Psychiatric Association [APA], 1987) did not find any changes in REM sleep, but did find modest correlations between movements in REM sleep and hyperactivity/impulsivity (Stephens et al., 2013). Recently, it has been shown a REM sleep overdrive in un-medicated children with ADHD which did not correlate to any psychopathologies or impaired daytime behaviors (Virring et al., 2016). Notably, however, our patients and controls differed from those in the above studies in age, applied diagnostic criteria and medication. Hence, as proposed by Kirov and Brand (2014), differences in age, diagnostic criteria used and medication status could significantly contribute to controversial sleep PSG findings in youths with ADHD and their effect on daytime behavior. Although further studies are needed to clarify the role of neurobiological variables, the present findings show that the delayed and/or deviant developmental decrease in REM sleep might represent a risk factor for developmental psychopathology.

Despite of the present findings, several considerations warrant against overgeneralization of the results. First, since periodic limb movements in sleep (PLMS) and sleep-disordered breathing (SDB) are among the most common sleep disorders in ADHD (Cortese et al., 2009), their presence was not reported here, because our focus was on the associations between sleep stages and daytime neurobehavioral functions. Thus, the role for PLMS and SDB in our findings mandates future investigations. Second, the sample sizes used are relatively small and heterogeneous in terms of age, gender and medication status before study. However, none of the multiple regression analyses extracted age, gender and medication status as predictors. Third, while it would be helpful to supplement our results by providing data about presence of parasomnias, sleep hygiene and circadian rhythm disorder, the lack of sleep diaries and actigraphy precludes such observations. Last, the present results might have emerged due to further latent neuroendocrinologic variables, which might have biased two or more dimensions in the same or opposite direction (Steiger et al., 2013).

## Conclusion

Our results indicate an opposite impact of REM sleep on neurobehavioral functioning, depending on presence or absence of developmental psychiatric disorders. Thus, the development of REM sleep functions and the way how these relate to different kinds of children’s behavior later on seems to depend on different factors (e.g., genes, environment, adaptation, etc.) influencing the child’s brain maturation either to a normal or an aberrant neuronal system.

## Author Contributions

RK, TB, and AR: Substantial contributions to the conception and design of the work. RK, SB, and TB: Interpretation of data and drafting the manuscript. RK, SB, and TB: Statistical analysis. RK and TB: Data selection and matching the groups for age and gender. SB, TB, and AR: Final approval of the paper draft and agreement to be accountable for all aspects of the work.

## Conflict of Interest Statement

The authors declare that the research was conducted in the absence of any commercial or financial relationships that could be construed as a potential conflict of interest.
